# Understanding the Role of Stigma and Misconceptions in the Experience of Epilepsy in India: Findings From a Mixed-Methods Study

**DOI:** 10.3389/fsoc.2022.790145

**Published:** 2022-05-13

**Authors:** Kritika Gosain, Tannistha Samanta

**Affiliations:** ^1^Department of Humanities and Social Sciences, Indian Institute of Technology Gandhinagar, Gandhinagar, India; ^2^Department of Sociology, Flame University, Pune, India

**Keywords:** epilepsy, stigma, misconception, India, marriage, cultural models of health and illness, employment

## Abstract

It is surprising that although 12 million people in India suffer from epilepsy this remains a thoroughly under-researched area in the sociology of health and practice. We address this intellectual and policy neglect by reviewing the social, psychological and legal challenges governing the lives of people living with epilepsy (PWE) by paying particular attention to negotiations in arranged marriages and employment. Drawing on the analytical frameworks of the sociological study of stigma, critical race theory and paying attention to the cultural models of health and suffering, this study utilized a combination of (online) survey data (*N* = 100) and in-depth qualitative interviews (*N* = 10) with PWE and their families. The online survey was administered to map the level of awareness about epilepsy and its clinical management among the general population, whereas the in-depth interviews were conducted to understand the experience, self-perception and everyday struggles of those diagnosed with the condition. Findings from the survey on non-PWE suggest a general lack of awareness and fearful misconceptions around epilepsy related seizures. In-depth interviews with PWEs revealed concealment (of the illness) as a dominant coping strategy to attenuate the social alienation and rejection associated with epilepsy. Further, PWE participants reported persistent discrimination, harassment and prejudiced understanding of diminished cognitive capacities at workplaces as a result of cultural myths and popular representations of epilepsy-related seizures. The study also demonstrated the significance of institutional support groups in assisting PWE to cope with symbolic violence and forge solidarities. We conclude with reflections on the ethical dilemmas faced by medical practitioners while dealing with social-medical interventions of epilepsy treatment. Overall, results from this study undergird the significance to revisit the social-moral as well as legal frameworks that persistently restrict opportunities for PWE in India. In an attempt to reimagine inclusive futures regardless of disease, disability and affliction, we attempt to move beyond the biomedical gaze and instead privilege stories of individual personhood, struggles and aspirations.

## Background

“*If a man buys a male or female slave and before one month has passed, bennu (seizure) falls upon him, he (the buyer) will return him to his seller and the buyer will take back the silver that he paid.”*

*(Hammurabi Code, 1750 BCE, 278)*

The above quote, although written during the 1750 BCE, is emblematic of the social anxiety, stigma and fear that is commonly associated with epilepsy in contemporary India and beyond. As such, epilepsy derives itself from the Greek term “epilepsia,” meaning to seize or take control. Even though the word holds its genesis in ancient Greece, epilepsy as a condition was first mentioned in the above Hammurabi text. It finds similar expressions in other ancient religious texts including the Gospel that addressed epilepsy as “demonic or unclean or deaf spirit” (Mark 9:14–29). The Spartans washed their newborns in wine in order to see if they were prone to epilepsy or sickly.^1^ In that case they were to be discarded (Stol, 1993), underscoring the fact that early expressions of this neurological condition were proffered in the language of mistrust, and impending misfortune. It is perhaps no surprise why present-day perceptions of the condition are clouded in prejudice in a context where social-medical awareness remains very limited.

Epilepsy, a largely neurological condition, is an acutely under- researched field in India. Epilepsy also remains a highly misunderstood condition globally with a cloud of confusion about its symptoms, risks and treatment. It affects about 570 million people worldwide with nearly 80% living in low- and middle-income countries (World Health Organisation, 2019). Epilepsy is a chronic health condition which for long has been stigmatized due to the physical manifestation of the condition. Social-psychological research on stigma has persistently demonstrated that it can lead to other health disadvantages among those that are being stigmatized (Hatzenbuehler et al., 2013, pp. 813–821). According to a report published by the World Health Organization (2001), “It has been estimated that 80–90% of people with epilepsy in developing countries are inadequately treated. Fear of stigmatization, cultural beliefs, lack of knowledge and low levels of education are factors that contribute to the treatment gap,^2^ resulting in people with epilepsy and related conditions not seeking treatment (Shorvon and Farmer, 1988, pp. s36–s54; Meinardi et al., 2001, pp. 136–149; Scott et al., 2001, pp. 344–351; Coleman et al., 2002, pp. 378–383; Wang et al., 2003, pp. 1544–1545). The lack of adequate treatment can have adverse effects on not just the health but also the socio-economic wellbeing of the individual. Social isolation, higher levels of dependence on others, low educational performance, poor employment opportunities, loss of productivity, and personal injury are all intangible costs which should also be included in the estimate of the burden of epilepsy (Meinardi et al., 2001, pp. 136–149).

According to Hatzenbuehler et al. (2013, pp. 813–821) “Stigma influences population health outcomes by worsening, undermining or impeding a number of processes that exacerbate poor health.” Stigma is an outcome of a structural procedure which begins with the labeling of an individual or a group of individuals. Stigma offers a fruitful framework to understand the experience of people with epilepsy (or PWE, henceforth) whose lives are commonly stereotyped as being in “possession by evil spirits” or “the condition being contagious” with respect to epilepsy.^3^ While anthropologists have offered cultural explanations of epilepsy that straddles the uneasy ambivalence of being gifted with supernatural powers and a construction of “epileptic (pathological) personality” (Reis, 2001, pp. 355–382), recent quantitative studies have shown depressive symptoms to be highly prevalent among PWE associated with elevated levels of social stigma and poor quality of life (Tombini et al., 2020).

This study relies on primary data collected through online surveys and interviews with PWE (associated with a non-governmental organization working on generating awareness and offering peer guided support on epilepsy) by paying particular attention to life course events including romantic relationship formation, marriage and employment opportunities. Since childhood epilepsy that continues till adulthood can affect a person living with the condition in a myriad of adverse ways, this study interviews PWE on their experience of seeking a partner, forging a marital alliance or pursuing a career of their choice. In what follows, this study presents scholarship on epilepsy and the social-psychological challenges faced by PWEs globally while underscoring the acute neglect in studying epilepsy in the social sciences in India. This study utilizes noted sociologist Erving Goffman's (1963/2009) conceptualization of stigma and critical race theorist, Crenshaw's (1989) seminal contribution to understanding the multiple axes of oppression and discrimination faced by PWE in India. This theory-driven understanding of this affliction is significant since the statistics on epilepsy and its social consequences in India is appalling: 90% of PWE are not treated, and 50% of them do not have access to drugs (World Health Organization, 2001). Additionally, the burden of disability from epilepsy has been long undetermined receiving limited attention in both medical and intellectual discourses. This neglect is more pervasive in the developing world. In one estimate, close to 90% of the worldwide burden of epilepsy is found in the developing world, with more than half occurring in the 39% of the global population living in countries with the highest levels of premature mortality and lowest levels of income (WHO, 2006). These alarming statistics also typifies the Indian experience.

To be sure, through a careful documentation of first-hand accounts of those living with this condition, this study emphasizes that discrimination as a result of epilepsy can begin very early on in one's life. According to a study based in India, seizures in the classroom can act as a trigger for negative responses toward the individual in the form of unequal treatment such as facing challenges in making friends, discontinuation of studies due to difficulty in learning as a result of the medications for epilepsy (Thacker et al., 2008, pp. 684–690). Additionally, due to the stigma associated with the condition, job opportunities remain limited for PWE (Jacoby et al., 2005a,b, pp. 1978–1987). Furthermore, a sustained fear of embarrassment from sudden seizures prevents PWE in maintaining friendships and romantic relationships in their adult lives. A similar sense of shame is a common sentiment among the family members of PWE who in turn often resort to concealing the condition in an effort to attain social acceptance (Singh et al., 2016, pp. 242–247).

Finally, it is important to note that public images or societal (mis)understandings of an enduring affliction (here, epilepsy) are often known to redefine the sense of self among people suffering from it. Anthropological interventions (Estroff, 1993, pp. 247–286) have reminded us that in the case of what is commonly known as “I am” illnesses: conditions that redefine the sufferer in socially significant ways and hence renders a morally stigmatized condition as opposed to other chronic conditions (or, “I have” illnesses) such as diabetes or hypertension. As such, anthropology's emphasis on stories or “illness narratives” of sufferers and their family members have allowed not only the possibility of social-moral justification for continued hope and care-seeking, but also for researchers to develop a phenomenology of chronicity (see Estroff, 1993; Good et al., 1994; Das, 2015). Henceforth, our analysis pays attention to the cultural models of suffering (Shweder, 2008) that allowed us to appreciate that the meaning of “health” is not unitary and its indexes vary by cultural understandings of intuitions, images and corporeal vulnerabilities. This aspect of suffering is particularly significant in understanding epilepsy since it is known to challenge the binaries of disabled and non-disabled as PWE move between episodes of seizures and periods of remission (Nair, 2019).

However, given the focus on the awareness (captured through an online survey) and experience of the condition (based on a qualitative questionnaire administered on PWE), the research design for this study was governed more by pragmatic factors of treatment than the anthropological symbolism of the condition. We acknowledge the risk of neglecting the conceptual conflation of diagnosis and identity that anthropologists have pointed out and include a brief discussion in the final section of the article.

## Stigma: Theoretical and Empirical Considerations

Sociological scholarship defines stigma as a label, a “spoiled” social identity different from the actual social identity (Goffman, 2009; Lim and Tan, 2014). While offering a formal theoretical construction on stigma, Canadian sociologist Goffman (1963/2009) describes it as a “…phenomenon whereby an individual with an attribute is deeply discredited by his/her society is rejected as a result of the attribute. Stigma is a process by which the reaction of others spoils normal identity” (Goffman, 2009, p. 3; Jacoby, 2008). This attribute is a result of discrepancy between the virtual and actual social identity, that is, the person they might be and the person they are. Goffman further notes, “While the stranger is present before us, evidence can arise of his possessing an attribute that makes him different from the others in the category of persons available for him to be of a less desirable kind….He is reduced in our minds from a whole and usual person, to a tainted discounted one” (p. 47). Goffman's theoretical articulation finds practical resonance with the experience of those diagnosed with epilepsy. Our results suggest that the lifelong stigma attached to the condition is a constant reminder for the individual of her/his marginal and devalued status in the society.

Intersecting stigma occurs when people are “marked” with multiple stigmas (Hargreaves et al., 2015). We argue that those suffering from epilepsy and seizures can become victims of symbolic violence and moral abuse in a context that holds prejudiced understanding of the neurological condition. Drawing from Crenshaw's (1989) seminal work on intersectionality, one can argue that PWE get caught in multiple “interlocking forms of oppression” that devalue their social identities. This can become particularly acute for women living with epilepsy (WWE) who are doubly disadvantaged due to their marginal social location. Our interviews with WWE seeking marital alliances reveal that since patriarchal ideologies create (arranged) marriage and motherhood to be socially integral part of the idealized feminine constructions, the lives of WWE get circumscribed within the interlocking forms of disadvantages. That is, the disadvantages that the WWE faces in India is not just a determinant of the misconceptions attached to the disorder/condition but also owing to their gender and social class position. This is compounded by the social anxieties of carrying out successful reproductive roles. Studies conducted in India suggest, “There is a misconception that WWE have difficulties conceiving, having healthy babies, raising children and that epilepsy worsens during pregnancy, while the majority of WWE have a normal pregnancy and delivery, an unchanged seizure frequency and around 93% chance of a healthy baby” (pp. 1–4). As such stigma can affect a range of health outcomes by worsening, undermining, or impeding a number of processes, including social relationships, resource availability, stress, and psychological and behavioral responses, exacerbating poor health (Hatzenbuehler et al., 2013, pp. 813–821). More recent scholars such as Stangl and Earnshaw (2019) focus on health-related stigma frameworks exploring psychological pathways at the individual levels; e.g., individuals experiencing stigma and those perpetuating it or both (Stangl et al., 2019).

Other noteworthy theoretical contribution to the understanding of everyday stigma is by American sociologist, Giddens (2010), who explains this process by stating that “Stigmatizing groups are one way in which society controls their behavior. In some cases, the stigma is never removed and the person is never fully accepted into the society” (Giddens, 2010, p. 269). In addition to this, stigmatizing specific health conditions can result in individuals withdrawing from certain social engagements. Michael Bury (2009) tries to interpret these consequences (of social withdrawal) by tracing them along the lines of chronic illness where he points out how societal outlook toward the individual results in bringing “the individual, their families, and wider social network face to face with the character of their relationships in stark form, disrupting normal rules of reciprocity and mutual support” (pp. 1–17). Significantly, stigma not only affects physiological outcomes but can also interfere with one's perception of his/her body and self-image. In an earlier research, Teal and Athelstan (1975) on sexual functioning in patients with spinal cord injuries, these researchers showed how patients' psycho-social considerations offered a useful definition of body image that is tied to psychological experiences and its affective states. A research study showed how adolescent children with epilepsy are influenced by the disease no matter what type of epilepsy, seizure control, or age of onset. These result in a reduced sense of trust about their bodies and themselves often causing increased emotional distress (Viberg et al., 1987, pp. 542–546).

Perhaps the most ironic part of this condition is that that unlike many other chronic, lifestyle altering diseases such as hypertension, diabetes or cardiac disorders, the symptoms and signs of epilepsy are unhidden (Ahmad, 2011, pp. 1–14). By losing control of themselves, PWE are seen as reverting to primitive acts and hence represent “anomic” terror to those without epilepsy (Bagley, 1972, pp. 32–45). These factors in turn can be source of anxiety and frustration for those suffering from epilepsy in a context where awareness is abysmally low and misguided notions about the condition are abundant.

Finally, given that most research on epilepsy adopt a biomedical narrative, we privileged our in-depth qualitative interviews to emphasize the “local moral worlds” within which individuals experience epilepsy. We contend that a socio-somatic model is significant to understand the social pathology of this condition that is often concealed or rendered invisible. Seen this way, our theoretical standpoint is aligned more toward Kleinman (1986) and Kleinman et al. (1995) creating a local ontology of illness that involves the roles of the family and the community (i.e., the social course) than the more individuated, “Western” biomedical course of illness and disorder eliciting a clinical prognosis. By doing so, we build on previous ethnographic studies from low-income countries that explore the embodied meanings of epilepsy and its social implications on marriage, employability and overall quality of life (Jacoby et al., 2008; Luhrmann et al., 2015). This standpoint, however, remains rare in the epilepsy discourse in India. Noteworthy exceptions include (see Thomas and Nair, 2011; Nair, 2019) who in her poignant autoethnography documented “how the disruptive, punctuated nature of epilepsy has the power to influence families and their worlds” (p. 188) with particular reference to marriage and the social concealment of the neurological condition.

Taken together, this study addressed three interrelated questions (the associated study methods deployed to examine these questions are indicated in parentheses):

1. What is the general level of awareness about epilepsy and related stigma among non-PWE population? *[captured through an online quantitative survey]*
2. How stigma and discrimination are experienced at multiple levels across the social institutions of marriage, employment and legal systems by PWE? *[qualitative, in-depth interviews with PWE and their family members]*
3. Given the context of a serious neglect of academic as well as public discourse on epilepsy, what are the (coping) strategies that are utilized by PWE? In the process of this examination, the study pays particular attention to the dimensions of social capital (a shared sense of being) mediated through non-governmental organizations working on generating awareness and support for those living with epilepsy *[qualitative, in-depth interviews with PWE and their family members]*.

## Epilepsy in India: a Review

Epilepsy continues to affect a significant population in India. Almost 12 million people account for almost one-sixth of the global epilepsy population” (Amudhan et al., 2015, p. 263). Epilepsy in India remains peripheral to the mainstream discourse on health and wellbeing. According to a recent document addressing the legal rights of People with Epilepsy in India, it was stated, “Despite good advances in medical therapy, Persons with Epilepsy (PWE) still face issues which seem to thwart their normal lifestyle. While social issues form a major part of their impaired lifestyle, they face many legal hurdles making their overall life more difficult than normal individuals” (IEA, 2017).

Similarly, in some regions of India, epilepsy is confused with a mental illness and the law equates epilepsy with temporary insanity till recently (Mani, 1997). According to the *Hindu Marriage Act of 1955* and the *Special Marriage Act of 1954*, it was stated that, a marriage under these Acts can be solemnized “if at the time of marriage, neither party suffers from recurrent attacks of insanity or epilepsy.” It wasn't until 1999, after a legal battle of 12 long years, Dr. K. S. Mani, often referred to as “father of Indian epilepsy,” and the IEA (Indian Epilepsy Association) were successful in getting the word “epilepsy” deleted from this law. Epilepsy related stigma accompanied by such laws have acted as a catalyst for PWE experiencing a sense of alienation and rejection especially in the case of marriage. In relation to marriage and parenthood, better psychosocial adjustment in many ways increases the possibility of a patient suffering from epilepsy to get married and make the decision about the offspring (Dansky et al., 1980, pp. 261–271). Unlike the industrialized countries, the concept of support groups for people with common medical problems continues to be rare in India. This becomes particularly difficult for those diagnosed with epilepsy to accept since they believe in avoiding disclosing their diagnoses for the fear of social stigma and the discrimination that is likely to follow (Varma et al., 2007).

## The Study: Recruitment Strategy, Data and Methods

We carefully mined secondary scholarship across a range of social science disciplines to develop the framework for this pilot study as well as the instruments that were utilized to collect primary data. Our study adopted a mixed-method (Creswell and Clark, 2017) framework in two- stages. In particular, borrowing Creswell's (2003) guide on mixed method strategies, the study followed a sequential study design: quantitative survey followed by qualitative data collection process. The recruitment strategy for both the quantitative survey and the qualitative interviews was guided by a purposive sampling technique given a general lack of awareness as well as the difficulty in identifying participants suffering from this condition. The semi-structured online questionnaire, modeled around the KAP^4^ approach was used to map the general levels of awareness and knowledge of stigma associated with epilepsy among the non-PWE population. The online survey began with questions pertaining to the levels of awareness about epilepsy in the general population followed by the social perception of PWE. This survey was shared and administered through social media platforms by the first author requesting voluntary participation in the study. Although effort was made to ensure heterogeneity in terms of age, occupational/educational profiles, gender identities and social classes, due to our reliance on social media, the participants in the quantitative part of the study primarily comprised of English-speaking young adults from middle to upper-middle class backgrounds. Consent was sought at the start of the digital survey. A total of 100 participants [including PWE (*N* = 10) and non-PWE (*N* = 90)] across the urban centers of Delhi, Mumbai, Gandhinagar and Pune took the online survey. In the later section of the paper, we use descriptive statistics to examine the results from the quantitative survey.

The goal of the qualitative interviews was to understand the PWE participants' life histories, reflections and perspectives around epilepsy and this formed the text data for the analysis. We utilized thematic analysis to delineate themes that emerge from this phase. The sample size (of 10) for this phase of the study was determined by theoretical saturation. While acknowledging that saturation is a subjective exercise, we followed Glaser's (1978) suggestion of data saturation, namely when “no new properties emerge and the same properties continually emerge” (p. 53). The qualitative interviews with PWE and their family members were conducted by the first author between November 2019 and March 2020 at the research site-*Samvedna Foundation* (SF, henceforth)- a support group working for PWE, based in Pune city, in the western Indian state of Maharashtra. Narrative-style interviews started with requesting participants to narrate their experiences growing up with epilepsy and then gradually asked to share more information about any stigma or discrimination that they might have faced while seeking romantic partners, in arranging marriage alliances or any work-place harassment on account of their neurological condition. These meetings with the PWE were timed during a matrimonial alliance meet organized by SF for PWE who routinely face stigma and harassment while attempting to seek arranged marriage proposals (Santosh et al., 2007, pp. 1007–1010). Overall, the first author spent about 30–40 h engaging with the study participants and also in participant observation at the SF meetings.

Findings emerging from the two phases (quantitative and qualitative) have been connected in the discussion stage of the study (Ivankova et al., 2006, pp. 3–20).

### Inclusion Criteria

Participants aged 19 and above, regardless of their gender identities and disability status, with at least a higher secondary degree and proficiency with taking internet-based surveys in English were included for the quantitative part of the study. For the qualitative phase, participants aged 20 and above, regardless of their gender identities, and those who reported being diagnosed with epilepsy for more than a year were included. Due the language familiarity of the authors, only those participants who spoke Hindi or English were included for the qualitative part of the study.

### Exclusion Criteria

For both phases of the study, we could not include those participants who were proficient in languages other than Hindi or English and those who had chronic disability or diagnosed cognitive impairment. Since our focus was on understanding perceptions and experiences of living with epilepsy, we considered these chronic health conditions to elevate psychological stress and stigma and hence a possible confounder to our analytical models.

### Analytical Strategy

Online survey results were analyzed using descriptive statistics followed by a chi-square test to examine the association between awareness of epilepsy and selected socio-demographic characteristics. The statistical significance level was set at α = 0.05. All bivariate tables were generated in STATA version 11.0® For the qualitative part, the rationale guiding the identification of themes were borrowed using thematic analysis (Braun and Clarke, 2006) guidelines while the interpretation of in-depth interviews and subsequently, narratives were guided by Allan Feldman's (1999) work on “*Conversation as Methodology in Collaborative Action Research*” (pp. 1–22).

### Research Ethics

This study was part of the first author's MA thesis and it had received ethical clearance from the Institutional Research Board of the Indian Institute of Technology, Gandhinagar.

Interviews were conducted by the first author using a combination of Hindi and English languages, after seeking (written) informed consent from the study participants. To ensure that the interviews dido not trigger emotional injury among the participants, the study first conducted the interviews through a mixed-gender focus group discussion among PWE and then followed up with individual interviews with interested participants. All names have changed (this study uses pseudonyms) to protect the privacy of our participants.

Table 1 presents socio-demographic characteristics of respondents who participated through the online questionnaire as well as in-depth personal interviews. Most of the participants interviewed for this study were in the age group 20–45 years (Mean ages: Male: 47 years; Female 52 years).

**Table 1:** Socio-demographic characteristics of the pilot study.

**Online questionnaire**	**Face-to-face qualitative interview**
**Sample size** 90 participants (non-PWE) Male: 44 Female: 45 Do not prefer to disclose: 1 **Age group** Mean age: 24 yrs 19–25 yrs: 74 25–30 yrs: 13 30 and above yrs: 3 Average years of education: 16 yrs (graduate degree and above)	**Sample size** 10 participants (PWE) Male: 3 Female: 7 **Age group** Mean Age: 30 yrs 20–30 yrs: 7 30–40 yrs: 2 50–60 yrs: 1 Average years of education: 16.75 yrs (graduate degree and above)

## Findings

### Online Survey (Non-PWE, *N* = 90)

A significant majority (87%) of the survey participants had knowledge of what epilepsy was and what were its possible causes (neurological condition; 83.7%). In terms of the source of information around this condition, survey results showed that social/print media to be the dominant (42%) form. Surprisingly, even though a significant population agreed to having heard about epilepsy and what caused it, majority of this population (74%) was not aware that epilepsy is treatable or controllable.

More than half of the online participants (54.5%) (*p* ≤ 0.01) noted of being aware of the social discrimination that PWE typically face. Additionally (39%) (*p* < 0.05) of the sample population were of the opinion (responded “yes”), that PWE are more likely than non-PWE to face challenges in securing a job as well as at workplaces due to their medical condition. Similarly, approximately (35%) (*p* < 0.05) of the participant population believed that it was indeed a challenge for PWE to find a non-PWE partner for marriage. Certain gender differences in responses are worth noting. For example, our online survey revealed that 78% of women participants noted that PWE face challenges in finding a non-PWE partner; the corresponding statistic for men is about 59%. This difference in perception could be attributed to the existing patriarchal ideologies that are known to restrict women's opportunities (in labor market as well as partner selection choices) thereby creating unequal social realities for men and women in India.

**Figure 1:**
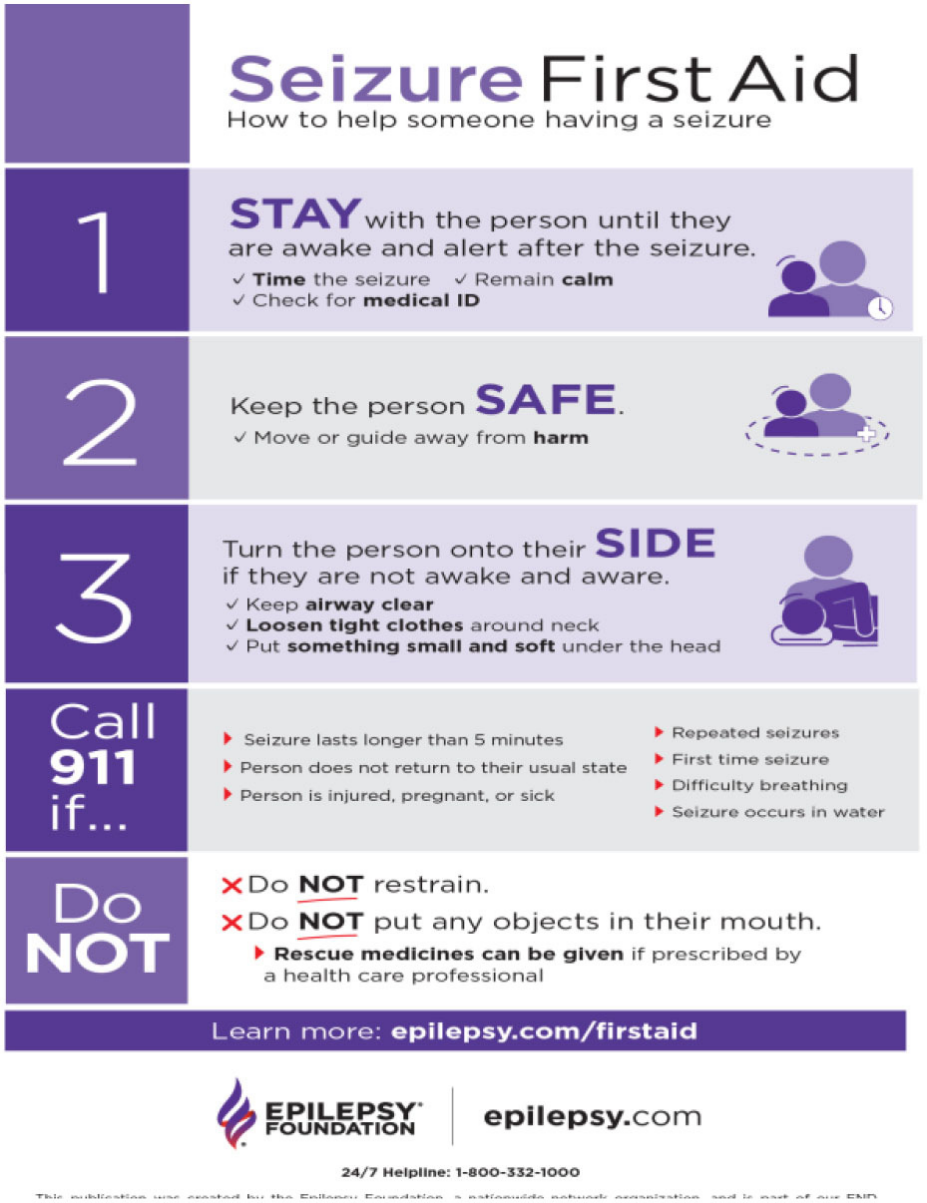
Epilepsy Foundation of America (2020) campaign best practices flier.

Since existing scholarship has noted the link between epilepsy related seizures and misconceptions, the current study attempted to understand non-PWE's awareness and knowledge about best practices in the event of a seizure experienced by a PWE. The following chart (adapted from, The Epilepsy Foundation, United States of America, 02/2020), describes the best practices (Figure 1).

The responses received regarding the practices or actions undertaken by the Non-PWE population as a response to any individual having a seizure induced due to epilepsy were quite shocking. The following Table 2 showcases the recorded responses that have been divided into three categories with reference to their nature of expression.

**Table 2:** Open-ended responses of online participants sharing observations in the case of witnessing a seizure (number of participants in each category is indicated within parentheses).

1. Misinformed responses (17 participants)	1. Placing a “clean cloth” or “handkerchief” to avoid the individual from biting his or her tongue was important. *Correct information*: The misconception of the person experiencing the seizure would end up biting their tongue can often result in hurting the individual. Doing so, can injure teeth or the jaw. A person having a seizure cannot swallow his or her tongue*^a^*.
2. Well-informed responses (6 participants)	1. Staying with the individual until the seizure subsided. 2. Loosening the clothes they were wearing, removing sharp objects from around the individual, placing a cushion under their head and positioning them to one side of their body.
3. Myths/superstitious responses (4 participants)	1. Making them (PWE) smell “smelly socks as it helps” 2. Making them (PWE) hold “iron keys” 3. “Stick a sock” inside the mouth of the individual.

a *Centers for Disease Control and Prevention, https://www.cdc.gov/epilepsy/managing-epilepsy/index.htm*.

Overall, the findings from the online survey show that although participants are familiar with the condition and are more or less sympathetic to those suffering from epilepsy, the participants' knowledge in understanding the treatment protocols remain very limited. Instead, their response in assisting someone having a seizure at a public location is guided by misconception and fear. These findings from the online survey are strikingly similar to Jacoby et al. (2008)'s study on the social life of epilepsy in China and Vietnam. The authors note that although participants in their study recognized epilepsy as an outcome of neurological events, there was considerable cultural ambiguity around its causes, experience and treatment. Overall, the authors conclude how knowledge and awareness of epilepsy present a complex interweaving of traditional cultural practices, myths, folk medicine and western biomedical systems of treatment.

In what follows, we use thematic analysis (Braun and Clarke, 2006) to record overlapping themes after transcribing the qualitative interview data. Once dominant themes were identified, we used theory and disciplinary knowledge to guide the use of the interview data. Following Braun and Clarke's (2006) contention of a flexible, inductive process, our theme development was largely an interpretive one than relying on pre-existing codes.

## Findings

### Epilepsy, Non-disclosure and Marriage

Family sociologists have persistently shown (parentally) arranged marriages (Kaur and Dhanda, 2014, pp. 271–292) as the most dominant form of partner selection process preserving caste, social class and religious hierarchies in India. For example, researchers Kaur and Dhanda (2014) show that even with the intervention of technologies in the marriage market (e.g., through the matrimonial websites), this apparent “modernity” allowing an expansion of choices does not radically change the cultural imaginations of marriage. That is, they contend that despite technology-mediated, these marriages remain circumscribed within the traditional boundaries of caste, social class and religion-typical parameters that parentally approved arranged marriages embody. By one estimate, it has been suggested that “Over 95% of marriages in India, Pakistan and Bangladesh are arranged (Rubio, 2014). Since epilepsy is often met with fear, misconception and mistrust, studies show that in contexts that rely on the cultural practice of socially sanctioned, parentally approved marriages, epilepsy can lead to poor prospects, poor marital quality and deficient self-management of the condition (Singh et al., 2016, pp. 242–247). Even though the condition affects the marital status of the individual irrespective of their gender, academic and medical research on epilepsy in India have pointed out that women are more likely to face discrimination in finding a suitable partner for themselves for marriage due to their condition. Sharma (Sharma et al., 2018, pp. 82–86) cautions that “Some of the important issues concerning women with epilepsy are perceived and enacted stigmas, management of epilepsy when the information is concealed from in-laws during marital negotiation, management of epilepsy during pregnancy, and its complications” (pp. 81–86). In Nair's auto ethnographic account (Nair, 2019) of encountering epilepsy both as a “patient” and as a “researcher,” she writes evocatively how for many of her respondents, divorce was a more stigmatizing label than epilepsy. Nair noted how her participants believed that epilepsy could *still* be “managed” through marriage but in case of a divorce, no social recovery was possible except through remarriage. For Jacoby et al. (2008), the intersection of prejudice, misconception and social norms created restricted opportunities for marriage among PWE in China and Vietnam. The authors noted that for many participants, the belief that epilepsy was inheritable and could be passed on to any offspring, resulted in the lack of marriageability of PWE.

These anxieties are revealed by participants of our study as well. The women participants expressed their concerns regarding the discrimination faced by them in finding a suitable marriage partner.

As Shilpa (sister of a participant diagnosed with epilepsy) commented,

“*My father does not allow my sister to tell people that she is suffering from epilepsy. We told him to not conceal my sister's condition when talking to a prospective proposal. Despite that he told my sister to tell the people who had come to see her that she has a “migraine” condition. They rejected our proposal. Now my father is hell bent on never disclosing that my sister has epilepsy. He said “what good did it (disclosing) do to us?”*

The account is one of the many experiences that are a testament to not just the difficulty faced by PWE in finding a suitable marriage partner but also the ways that are adopted by them to cope with the anticipated rejection from the society. Goffman (1963/2009) describes the role that society plays in establishing the definitions of “normal” and the “Other.” The “Other” is associated with some form of prejudice that socially devalued an identity. To understand the stigmatized identity as a result of epilepsy, we find Goffman's concept of “concealability” a useful tool especially in the context of arranging marriages among PWE. As pointed out earlier, Goffman suggested that individuals without visible signs of disability are likely to “pass” as “normal” by initiating behaviors or techniques that would act as a camouflage of the disability. The above provided testimony is an example of how PWE are often compelled to resort to concealment as a way to seek social acceptance through marriage proposals.

Related studies such as Singh et al. (2016, pp. 242–247) articulate this social dilemma: “PWE fail to disclose the fact that they have epilepsy during matrimonial negotiations while those who disclose upfront are often faced with rejection. Professionals in epilepsy care frequently face the challenging task of providing counsel regarding the optimal way to deal with the situation” (p. 4). As discussed earlier, the phenomenon of concealment is a consequence of the enacted stigma associated with epilepsy which comes in the form of breakdown of matrimonial negotiations and in extreme cases, divorce. In India, the Hindu matrimonial statute equates epilepsy with insanity, both being grounds for divorce prior to 1999 (Pathare et al., 2015, pp. 7–13) (p. 7). Concealment of the condition would often act as a ground for divorce.^5^ Overall, our findings on concealment as a strategy deployed by PWE and their families is similar to those found in Singh's (2016) study that show how non-disclosure of the condition during marriage negotiations continues even after marriage such as replacing epilepsy pills with vitamin pills that has serious consequences on self-management of the PWE.

Yet another participant recalls her experience with disclosing the condition and the lack of awareness about epilepsy among people. Anita (has accompanied her sister to the SF meet) laments that lack of awareness and misconceptions around epilepsy.

“*We told them that she (respondent's sister) suffers from epilepsy. They didn't understand at first. Then we had to explain that she suffers from “fits”. They instantly refused the proposal. It's frustrating at times people do not understand that epilepsy is merely a neurological condition which is perfectly treatable or can be kept under control.”* She continued*, “But we definitely never conceal the fact that our sister has the condition. It is important to tell because she needs to take her daily medications daily.”*

Since caste remains an important determinant in arranged marriages, PWE face the double burden of first, being rejected due to their condition, and second, attempting to select a spouse within their own caste groups. As a result of this dual burden, the pool of potential spouses is highly restricted for PWE. One of the interviewees pointed out how they gave up a suitable match for their sister due to caste-based restrictions.

As Rekha (who accompanied her sister) explained,

“*The person was a Maharashtrian. A NRI. He also had the same condition (epilepsy). We are Punjabis. Bhatias. My father refused the proposal. Caste is too important for him. Since then it has been difficult to find a suitable match for my sister. My father told me and my husband to re- approach Samvedna Foundation enquiring on whether the proposal was still available. But now that person is married.”*

Curiously, the lack of awareness and fear associated with the condition cannot be attributed only to those who are not suffering from epilepsy (i.e., the Non-PWE) but to the PWE themselves. For example, one of our participants, who had come for the matrimonial meet to seek a partner for herself, noted

“*It's better not to get into a relationship or marriage because it (epilepsy) may be genetic and may pass on to the next generation” (Megha, 28 yrs.)*.

The response also underscores the loss of self-esteem that PWE experience as a result of the recurrent discrimination faced by them due to their condition. These findings are akin to previous studies on marriage and epilepsy that show the effects of poor Quality of Life and reduced satisfaction as a result of challenges in partner selection and support (Agarwal et al., 2006, pp. 409–415).

### Epilepsy and Institutional Discrimination

As noted in our review of scholarship, studies have shown that PWE are more likely to face institutional discrimination than others in comparable economic opportunities. Even though the condition can be controlled with AED (Anti-Epileptic Drugs), yet the challenge for PWE to get a preferred job persists. The narratives collected from the interviewees were a precedent to the discrimination faced by people with epilepsy. According to Tanya *(54 yrs)*,

“*Yes it might be difficult for PWE to get a good job. I have to live with the fear of getting seizures anywhere. It could be at work, at somebody else's home, at social gatherings. It's very embarrassing when it does happen.”*

We noted that some narratives are more hopeful when it comes to employment than fear-based discrimination in seeking marital alliances. For instance, one of our interviewees who had accompanied her sister to the SF meet, observed

“*My sister is a teacher. Her colleagues are well aware of her condition. They know how to handle her in case she has a seizure.”*

The laws regarding epilepsy with respect to certain government and administrative positions also affect the morale of an individual suffering from the condition. For Nitin it was a hard decision to opt for engineering. He observed,

“*I had to give up my dream of being a part of the defense forces because of my condition.”*

Driving for those diagnosed with epilepsy has been a contentious issue in many countries including India where PWE are routinely disallowed to undertake such tasks (Tripathi et al., 2012). This law is despite scientific evidence suggesting otherwise. For example, a recent review article argued that a seizure during driving is dangerous, but the risk is somewhat predictable and is not substantially higher than for those with other chronic medical conditions, such as heart disease, diabetes or even for those who consume alcohol (Sheth et al., 2004, pp. 1002–1007). These legal challenges that discriminate PWE were observed by several of our study participants.

Again, school systems in India and elsewhere also inadequately socialize children and young adults about this condition. A systematic review of knowledge and attitudes toward epilepsy among school teachers in 27 countries found deficits in teacher knowledge and negative attitudes were pervasive across all studies (Jones et al., 2018, pp. 59–68). Teachers do not receive formal training in epilepsy and several studies indicated that they lack adequate knowledge about seizures and confidence working with a child with epilepsy (Bishop and Boag, 2006, pp. 397–405; Hinton and Kirk, 2015, pp. 107–120). Consistent with this empirical finding, one of our interviewees' responses to facing challenges in socialization was shocking. According to Manju's mother, who herself is a medical practitioner,

“*People are really insensitive toward PWE. My daughter's epilepsy makes it a little difficult for her to communicate freely. People perceive that she has some psychological condition. I clearly remember how difficult it was for us to get a hostel room allotted for our daughter due to her condition. During the course of her study, she had a seizure and we came to pick her up. Once she recovered, we decided on taking her back to her college. On reaching we saw that all her belongings had been shifted outside and her room was locked. On asking what the matter was, the warden simply replied, “we cannot take the burden of looking after a student with such a condition.”*

Overall, the institutional biases and legal neglect described above are emblematic of the larger social bias and misconception surrounding this medical condition that is both treatable and can be safely managed with prescription drugs.

We conclude this section by describing the results emerging from our participants' reflections on psychological deprivation that is commonly associated with an epilepsy diagnosis. Most of our participants acknowledged that the diagnosis itself can have a deep impact on the psyche of the individual. But this impact is burdened further with the discrimination that PWE face as a result of epilepsy which often becomes a constant reminder of their “disability.” When asked about how society's outlook toward the condition affects them, one of the respondents shared her fear of embarrassment due to her recurrent seizures that could take place in public gatherings. In some instances, the interviewee's exhibited hesitation in directly admitting to the psychological pressure that epilepsy stigma had had on them. For instance, Sudha, one of the participants in this study, concluded, “*And you should try to live alone if you have epilepsy*.” This response that underscores self-rejection is a testimony of the fear and exclusion that many PWE face throughout their lives. This sentiment of an atomized living as a result of epilepsy was a common refrain among other participants who willfully rejected companionship. However, it can be argued that while self-disclosure elevates vulnerability and social gaze, it also allows PWE to build resilience especially among themselves. This aspect is capably articulated in Nair's work where she notes how her self-disclosure as someone who had been diagnosed with epilepsy opened up a previously sheltered space between herself (as a researcher) and her interlocutors. Perhaps, this also explains why support groups and organizations working to build solidarity are significant sites of building resilience among people with a shared sense of being.

However, only a handful of the participants reported affiliation with a support group or an organization that is focused on PWE. For the most part, people's perception of the condition with respect to marriage and employment continue to be governed by early cultural definitions of epilepsy making it a challenging task for PWE to integrate in mainstream society without facing prejudice and suffering a loss of self-worth. These findings resonate with recent studies on social-psychological implications of the condition underscoring that regardless of varying cultural beliefs about epilepsy, implications for stigma and discrimination remain a common denominator for PWE's everyday experience, globally (Tombini et al., 2020; Kwon et al., 2022).

### Epilepsy in Cultural Imagination

We conclude our analysis by reviewing certain cultural lexicon to describe epilepsy and related conditions. We contend that using strong and an abusive cultural vocabulary in everyday conversations and popular portrayals add to the stigmatized identity of PWE. As pointed out by Fernandes et al. (2009, pp. 1280–1283), “the words we use can influence our perceptions and have consequences for social stigma…The term “epileptic” tends to elicit more negative perceptions and a higher degree of stigma than does “person with epilepsy.” For example, in one of the interviews conducted during the research, a respondent pointed out how people did not “*understand*” the term epilepsy and would only make sense of the condition once it was addressed through the pathology of “*having* “*fits*.” Another common terminology used to define epilepsy in India is “*mirgi*.” These terms do not just define the medical condition but rather the physiological manifestation which can be deemed as socially violent and deviant. Often, these expressions are also presented in the language of mockery and scorn. For instance, the use of Hindi phrases such as “*pagal pan ke daure or daure padhna”* (to behave like a lunatic) is an example of the manner in which epilepsy has been received in our society. Epilepsy is often relegated to other worldly types of condition, where the individual is perceived to be unaware of one's own self- a profound lapse of memory and cognitive capacities. This perception discourages the individual suffering from this condition from seeking appropriate help due to the fear of being considered socially deviant or worse, being afflicted with insanity.

We note that popular representation of the condition may also play a role in shaping people's perception of epilepsy. As such, popular media theories, such as cultivation theory (Gerbner et al., 1980, pp. 37–47) and social construction theory (Higgins, 1987, pp. 319–340) emphasize the role of media images and portrayals on the viewer's perception and construction of reality. In the case of epilepsy, a “seizure” is often visually imagined as an afflicted body experiencing sudden, uncontrollable jerks, frothy saliva and a complete suspension of cognitive capacities (Wong et al., 2013, pp. 247–250). However, in reality, not all seizures in epilepsy include such physical manifestations and vary in their levels of intensity and types. Portrayal of epilepsy in the form of generalized convulsions in an overly dramatic and violent way involving certain specific traits listed above is routinely found in cinematic representations. For example, a recent release “*The After Party” (2018)* on the OTT platform Netflix, is a story about an aspiring young rapper diagnosed with epilepsy. The story highlights his struggle to make it in the music industry and how he gains notoriety when suffers a seizure on the stage while performing and the video of the event goes viral. The movie continues to portray the condition of the protagonist in a comedic light, with him being referred to as “seizure boy.” Kerson et al. (1999) analysis of characters with epilepsy in 20 English language films since the 1950s show that most characters are portrayed as sad, damaged individuals that are feared and often linked to psychiatric disease (Kerson et al., 1999, pp. 1163–1167). Although there seems to be shift in the way how epilepsy is viewed, say in recent YouTube videos where portrayals are more sympathetic and factually accurate (see Wong et al., 2013), but the social pathology of affliction and a lack of agency continue to inform such representations.

## Discussion: Understanding Epilepsy Related Stigma

This study demonstrated how cultural belief systems and misconceptions continue to govern society's attitudes toward people with epilepsy. Moving away from a biomedical perspective, we paid attention to the cultural models of illness and suffering to guide our understanding of the meaning-making process around a condition that remains stigmatized and non-disclosed. Utilizing a mixed-methods design-(online) survey data and in-depth interviews- our study revealed the interpersonal stigma experienced by PWE especially in marriage and labor markets. Our brief review of the legal frameworks also led us to suggest that several laws guiding civil rights (e.g., procuring driving licenses) are based on unscientific and prejudiced claims reifying the stigma associated with epilepsy.

The online survey results revealed that people with limited knowledge about epilepsy, or without personal contact with someone with epilepsy, report poorer attitude toward the condition and its treatment- an observation that has been persistently shown in previous studies (Guekht et al., 2007, pp. 128–133). Further, the magnitude of the negative attitudes is aggravated by the presence of misconceptions about epilepsy- as being a form of insanity, is considered untreatable, contagious, hereditary, a form of learning and cognitive disability (ibid). As such, consistent with prior studies (Aydemir et al., 2016, pp. 76–80; Bautista, 2017, pp. 7–11) respondents note that negative stereotypes promote concealment of the condition which in turn heightens internalized stigma. The social anxiety of rejection as a result of the condition is exacerbated among female PWE participants. Many of them suggested solo living (eschewing any meaningful companionship) as a coping strategy to avoid shame and social embarrassment. We speculate this perception among women is an outcome of patriarchal ideologies that restrict women's (more than men's) freedoms in seeking employment or a partner of their choice.

Again, more than half (54.5%) of the online survey participants agreed that PWE faced social discrimination. The in-depth interviews with PWE and their family members supported this observation when they reported the challenges involved while attempting to seek romantic partners, spouse selection through arranged marriages and at work places. Most of them attributed the epilepsy-related stigma and isolation as a result of the physical manifestation of the condition. It is worth noting that the role of organizational initiatives (here, SF) in forging solidarities and developing collective strategies to fight biases, was an important source of support perceived by the participants and their families. From the focus group discussion and the participant observation, it was evident that the support groups act as open and safe spaces for PWE to address their inhibitions without any fear of being socially judged and share their personal struggles and aspirations.

In case of employment, the study revealed that often at work places epilepsy is perceived as being a condition that interferes with cognitive capacities, while PWE participants shared that much of their quality of work is impacted by the reactions that they are faced with when their (PWE) peers and colleagues are a witness to their seizures. Self-pity and embarrassment about one's own condition were defining sentiments of the participants.

In the case of medical conditions such as epilepsy that are highly susceptible to stigma, the possibility of ethical conflicts is bound to be high. As noted earlier, of the 50 million people with epilepsy worldwide, around 80% reside in resource-poor countries, which are ill-equipped to tackle the enormous medical, social and economic challenges posed by epilepsy (Radhakrishnan, 2009, pp. 323–330; Shostak and Ottman, 2006). The ethical challenge presents itself in the form of misallocation of medical and social resources for bridging the high epilepsy burden (Tan and Avanzini, 2009, pp. 1975–1977). This situation is further exacerbated by social class lines (lower income groups have both limited knowledge and medical capital to afford effective treatment). In terms of medical research on epilepsy, there has been pleas in the scientific community to develop moral-ethical guidelines that provide financial, material and human resources to enhance the care of PWE in resource-poor countries (Tan and Avanzini, 2009, pp. 1975–1977; Radhakrishnan, 2009, pp. 323–330). Additionally, the medical practitioners often see themselves in a difficult position when asked about the treatment by the patient. The diagnosis not being disclosed prior to marriage (particularly in the case of WWE) can act as an ethical challenge for the doctors. A similar situation of ethical and legal concern arises in cases that are raised by genetic tests, generally, including appropriate informed consent, autonomy, confidentiality and privacy of genetic information, and the imperative of balancing individual, parental, and societal interests when considering genetic testing for a minor.

This study is however not without limitations. Owing to time and geographical constraints within which this pilot project operated, we were able to administer the online survey among urban, middle/upper middle-class participants who spoke and understood English (used in the survey questionnaire). We acknowledge that our (surprising) finding of a very high knowledge about the condition could be attributed to this sample bias. However, despite our sample population being educated, urban and middle-class, the high degree of misconceptions about the best practices around epilepsy is alarming. In a way, it is reflective of the public image of epilepsy that is clouded in stereotypes and fear. We are also aware that our framing of epilepsy as a public health concern demanding treatment and intervention could be problematic from the perspective of anthropology or to some extent medical sociology that view affliction as something inextricably tied to personhood and thereby altering the self and identity in enduring ways. While being fully aware of this ethnographic critique, we allowed our study design to be governed by practice-oriented factors focusing on the social experience of epilepsy and coping behaviors associated with it. As such, given our study design we are unable to make a commentary on the cultural shaping of the phenomenology of epilepsy. We also acknowledge that our goal of unpacking the local ontology of illness has not been fully explored since resources (time and funding) did not allow for multiple interviews with our interlocutors through which the cultural indexes and imageries would have been better examined. While acknowledging that this would have been a fruitful line of inquiry, we hope our study offers a useful starting point to advance our understanding of the complex anthropological symbolisms associated with affliction and social suffering.

Notwithstanding these limitations, this study makes an attempt to underscore the challenges-social, psychological, legal and to some extent, medical- that need to be addressed to help improve the quality of life of PWE in a context where knowledge about this condition is rooted in prejudice and fear. The study reveals that a person suffering from epilepsy is caught in the interlocking axes of disadvantages that limit economic as well as non-economic (romance, companionship and overall wellbeing) opportunities. We contend that unless positive socio-legal interventions are directed to improve both awareness and societal preparedness, lives of PWE are bound to be caught in shame and embarrassment for a condition that they have no control over. All in all, our study highlighted the importance of recognizing cultural variations in understanding a seemingly neurological condition. While the biomedical gaze governed the etiology and treatment of epilepsy, paying attention to the social trajectory of the illness allowed us to appreciate the lived experiences of felt and enacted stigma of the corporeal manifestations of the chronic condition. Seen this way, this study contributes to the small but significant body of work that attempts to establish a dialogue between the messy world of (qualitative) meaning-making with sanitized data (Luhrmann et al., 2015). Seen this way, our study is a plea to re-imagine inclusive futures that celebrates individual personhood regardless of disease, disability and affliction.

## Data Availability Statement

The original contributions presented in the study are included in the article/supplementary material, further inquiries can be directed to the corresponding author/s.

## Ethics Statement

The studies involving human participants were reviewed and approved by Institute Ethics Committee Indian Institute of Technology Gandhinagar. The patients/participants provided their written informed consent to participate in this study.

## Author Contributions

KG contributed in data collection, analysis, and writing the article. TS contributed in formulating the theoretical frameworks, research methodology, and writing the article. Both authors contributed to the article and approved the submitted version.

## Conflict of Interest

The authors declare that the research was conducted in the absence of any commercial or financial relationships that could be construed as a potential conflict of interest.

## Publisher's Note

All claims expressed in this article are solely those of the authors and do not necessarily represent those of their affiliated organizations, or those of the publisher, the editors and the reviewers. Any product that may be evaluated in this article, or claim that may be made by its manufacturer, is not guaranteed or endorsed by the publisher.

## Footnotes

^1^Plutarch, Life of Lycurgus 16, 3;cf. E.D Baumann, 186 f.

^2^The difference between the number of people with active epilepsy and the number of people who are being appropriately treated in a given population at a given point in time, expressed as percentage, is known as the “treatment gap” (Meinardi, 2001).

^3^We use the term PWE to denote the group that is diagnosed with this condition while being fully cognizant of the challenges and dangers of using a specific nomenclature that can further stigmatize the experience of living with this condition. Our decision to use this term is based on Reuber's (2014) editorial piece in *Seizure* where the author laments the lack of an official label that is respectful and one that appropriately guides research and practice.

^4^The Knowledge, Attitude and Practice (KAP) framework is a scale which is structured at highlighting the social condition of an illness in the society through the means of an “educational diagnosis” According to a WHO report on the status of epilepsy in Southeast Asian nations, he attitude toward epilepsy in India, Indonesia and Sri Lanka is far more negative than it is in developed nations. This is possibly related to the higher illiteracy rate. However, in Kerala, a southern Indian state, with a high literacy rate, the attitude is extremely negative, and epilepsy is generally believed to be a mental illness” (WHO, 2005).

^5^An example of a legal case that points out the lapse in the Hindu Marriage Act and how it has been unsuccessful in catering to the rights of PWE. One such case was Sudha Suhas Nandanvankar vs. Suhas Ramrao Nandanvankar on 15 September, 2004. As stated, “The applicant was married with the opponent on 21-5-1995 according to Hindu Laws. The said marriage has been annulled by a-decree of nullity dated 16-3-1996 on the grounds that the *applicant-wife was suffering from epilepsy at the time of marriage* (26).
